# Draft Genome Sequence of *Enterobacter cloacae* S23 a Plant Growth-promoting Passenger Endophytic Bacterium Isolated from Groundnut Nodule Possesses Stress Tolerance Traits

**DOI:** 10.2174/1389202924666230403123208

**Published:** 2023-06-23

**Authors:** Pavithra Ramakrishnan, Manikandan Ariyan, Anandham Rangasamy, Raghu Rajasekaran, Krishnamoorthy Ramasamy, SenthilKumar Murugaiyan, Veeranan Janahiraman

**Affiliations:** 1 Department of Agricultural Microbiology, Tamil Nadu Agricultural University, Coimbatore, 641003, India;; 2 Institute of Ecology and Earth Sciences, University of Tartu, Tartu, Estonia;; 3 Department of Crop Management, Vanavarayar Institute of Agriculture, Pollachi, Tamil Nadu, India;; 4 Agricultural College and Research Institute, Tamil Nadu Agricultural University, Eachangkottai, India

**Keywords:** Endophytic bacteria, whole-genome, PGPR, drought, salt stress, *Enterobacter cloacae*

## Abstract

**Aim:**

This study aims to reveal the passenger endophytic bacterium *Enterobacter cloacae* S23 isolated from groundnut nodules and to underpin the molecular mechanism and genes responsible for abiotic stress tolerance.

**Background:**

A variety of microorganisms that contribute to nodulation and encourage plant development activity in addition to the nodulating *Rhizobium*. Passenger endophytes (PE) are endophytes that accidentally penetrate the plant without any selective pressure keeping them in the interior tissue of the plant. PE possesses characteristics that encourage plant development and boost output while reducing pathogen infection and improving biotic and abiotic stress tolerance. However, there is a lack of molecular evidence on the passenger endophyte-mediated alleviation of abiotic stresses.

**Objective:**

This study was formulated to reveal the draft genome sequence of *Enterobacter cloacae* S23, as well as genes and characteristics involved in plant growth promotion and stress tolerance.

**Method:**

The data were submitted to PATRIC and the TORMES-1.0 Unicyclker tools were used to conduct a complete genome study of *Enterobacter cloacae* S23. The TORMES-1.0 platform was used to process the reads. RAST tool kit (RASTtk) was used to annotate the S23 sequence. The plant growth-promoting traits such as indole acetic acid production, siderophore secretion, production of extracellular polysaccharides, biofilm formation, phosphate solubilization, and accumulation of osmolytes were examined under normal, 7% NaCl and 30% polyethylene glycol amended conditions to determine their ability to withstand salt and moisture stressed conditions, respectively.

**Result:**

We report the size of *Enterobacter cloacae* S23 is 4.82Mb which contains 4511 protein-coding sequences, 71 transfer RNA genes, and 3 ribosomal RNA with a G+C content of DNA is 55.10%. Functional analysis revealed that most of the genes are involved in the metabolism of amino acids, cofactors, vitamins, stress response, nutrient solubilization (*kdp, pho,* pst), biofilm formation (*pga)* IAA production (*trp),* siderophore production (*luc, fhu, fep, ent, ybd*), defense, and virulence. The result revealed that *E*. *cloacae* S23 exhibited multiple plant growth-promoting traits under abiotic stress conditions.

**Conclusion:**

Our research suggested that the discovery of anticipated genes and metabolic pathways might characterise this bacterium as an environmentally friendly bioresource to support groundnut growth through several mechanisms of action under multi-stresses.

## 1 INTRODUCTION

Environmental stress is a significant impediment to sustainable food production due to the production of reactive oxygen species (ROS), which endanger cell organelles and macromolecules, such as proteins, DNA, and enzymes, and cause apoptosis, which in turn indirectly affects yield output. To achieve sustainable agriculture, synthetic agrochemicals and conventional agricultural practices must be replaced with Plant growth-promoting rhizobacteria (PGPR), which promote plant growth and stress tolerance. Plant growth-promoting bacteria (PGPB) are a collection of taxonomically unrelated bacteria that can form symbiotic relationships with plants to enhance their growth in adverse environmental conditions. PGPB can be found in epiphytic bacteria (which adhere to the leaves and roots) or endophytes (bacteria presents within plant tissue). PGPB controlled plant growth through a variety of mechanisms, including nutrient solubilization and mobilization, altering the levels of various plant hormones, and acting as an antagonist against various pathogens and insects [1]. Multiple species of the family Enterobacteriaceae exist in various environments and are related biochemically and genetically. Desert-isolated *Enterobacter* strains have been observed to be resistant to arsenic. [2], glyphosate degradability in Algerian desert soil [3] and the rhizosphere of oil-contaminated desert plant soils [4]. Plant growth-promoting (PGP) traits have also been stated in members of the genus *Enterobacter*. For example, *Enterobacter* sp. EnB1, which was isolated from semi-desert soil (Mexico) shown to be solubilized the insoluble phosphate [5], and *Enterobacter* sp. B6, which was isolated in Algerian desert soil, had been demonstrated to have biopesticide properties against *Locusta migratoria* nymphs [6]. We isolated *Kosakonia oryzae* ESB1 and *Enterobacter cloacae* S23 from groundnut nodules that possessed multiple plant growth-promoting traits and promoted groundnut growth under pot culture conditions [7]. There are several *Enterobacter* species and related members of the Enterobacteriaceae family that exhibit PGP in response to abiotic stress have also been identified in recent years. For instance, during salt stress, *E*. *cloacae* SBP-8 (formerly *Klebsiella* sp. SBP-8) produced systemic tolerance in wheat [8]. *E*. *cloacae* UW5, which was capable of producing significant quantities of indole-3-acetic acid (IAA) [9], or *E*. *oryzae* Ola 51T, has been reported as a nitrogen fixer in a previous study [10]. In addition, it has been reported that members of the genera *Enterobacter*, *Klebsiella*, and *Leclercia* synthesis siderophores, and antifungal compounds, solubilize insoluble phosphate, and fix atmospheric N_2_ [11]. In addition to the nodulating *Rhizobium*, numerous bacteria aid in the nodulation process and stimulate plant development. Passenger endophytes (PE) are endophytes that enter a plant by accident and remain there in the absence of forces that would maintain their presence. In a previous study [7], we carefully characterized the rhizobial and passenger endophytes in groundnut nodules and recorded their PGP. The capacity of endophytes to confer plant stress resistance opens a new avenue for mitigating the detrimental effects of various stresses on agricultural output. From the root nodules of *Lespedeza* sp., several passenger endophytes and *Rhizobium* (R) were isolated [12]. From the pigeon pea root nodules, 114 bacterial strains were isolated, of which nearly 60 percent were *Rhizobium* and the remainder were PE [13]. These PE have characteristics that improve plant growth, and yield, decrease pathogen infection, and increase the plant's resistance to biotic and abiotic stress [14]. Molecular evidence regarding the passenger endophyte-mediated alleviation of abiotic stress is lacking. Understanding the role of various key regulators, such as biochemical adjustment, genetic modifications, genes responsible for promoting plant growth, resistance to abiotic stress, and molecular strategies in abiotic stress alleviation, will aid in the development of environmentally friendly and sustainable agriculture. In this study, we present the draft genome sequence of *Enterobacter cloacae* S23, in addition to genes and traits involved in plant growth and abiotic stress resistance.

## 2 MATERIALS AND METHODS

### 2.1 Source of Strain

*Enterobacter cloacae* S23 (NCBI accession No. MT830893) was previously isolated from surface disinfected groundnut root nodules of VRI 2 variety (bunching type) by using the nutrient agar medium (7).

### 2.2 Growth Condition and DNA Isolation

*Enterobacter cloacae* S23 was grown in a liquid nutrient medium at 28 ± 2°C for 24 hours. The genomic DNA was obtained using the HiPurA^®^ Bacterial Genomic DNA Extraction procedure (Hi-Media, India). DNA was further analyzed using NanoDrop (Thermo Scientific, Nanodrop 2000c) spectrophotometer and subjected to WGS (whole-genome sequencing) through Illimina-Miseq platform.

### 2.3 Comprehensive Genome Analysis and Genome Assembly

*Enterobacter cloacae* S23's genome was analyzed using the PATRIC (Pathosystems Resource Integration Center) service. The TORMES-1.0 platform was used to process the reads. An average read length of 149 bp was left after quality trimming using default parameters in trimmomatic tool. SPAdes (version 3.11.1) were used for assembly, yielding 35 contigs with a contig length of 4824008 bp. The default parameters were used for all bioinformatics operations. Chromosome maps were generated using Proksee CG viewer.

### 2.4 Annotation of Genome Assembly

The genome annotation of *E*. *cloacae* S23 was done using the RASTtk to compare with other genomes present in the PATRIC server to identify the gene of interest, various functional characterization, and phylogenetic analysis. Various protein annotations such as protein’s function [15], Gene Ontology [16], KEGG pathways protein [17], and types of protein families [18] were done as per the cited protocols. Abricate and PARTIC servers were used to identify the virulence factor from the databases like Victors Data Base, VFDB, L, and PATRIC-VF. More than 90% of query coverage is retained. To comprehend the involvement of a collection of proteins that carry out a specific biological function or structural complex, a subsystem analysis of the genome was conducted. GC skew value was calculated using the online tool SkewIT (shinyapps.io).

### 2.5 Genome Comparison

We used the genomes or contigs aligned against each other using the tools MAUVE v 2.3.1. The average nucleotide identity (ANI) was used for phylogenetic tree construction and nucleotide similarity between the whole genome calculated in OAT v0.9. Published genomes were obtained from the PATRIC database (http://patricbrc.vbi.vt.edu), which was used for the alignment. To calculate pairwise similarity, the aligner’s sequence alignment file was processed. Briefly stated, we looked for conserved blocks in the alignment file and then focused on areas with 50 consecutive gaps to determine the similarity score based on pairwise sequence similarity percentage and coverage score, which represents the percentage of the genome that could be aligned pairwise.

### 2.6 Phylogenetic Analysis

Mash/MinHash46 was used to identify the nearest reference genomes. The PATRIC global protein families (PGFams) for these genomes were identified in order to evaluate the phylogenetic position of this genome. MUSCLE was used to map the nucleotides for each of these sequences to the protein alignment, and the protein sequences were then aligned. Concatenating the various amino acid and nucleotide alignments into a data matrix, which was then analyzed using RaxML and rapid bootstrapping to get the tree support values.

### 2.7 Plant Growth-promoting Traits and Endogenous Accumulation of Osmolytes

The plant growth-promoting traits of *E*. *cloacae* S23 under normal, 7% salt, and moisture stressed conditions (using polyethylene glycol PEG 6000) were determined. The synthesis of indole acetic acid was assessed using the technique reported by Patten and Glick [19]. *E*. *cloacae* S23 was inoculated into the test tube containing 5 ml of liquid nutrient medium added with salt (0%, 7% w/v NaCl) and 30% PEG 6000 (moisture stress) added with 0.1% tryptophan and kept for incubation at 28 ± 2°C for 2 days in a shaking condition at 120 rpm. The cells were separated by centrifuging at 12, 000 rpm for 10 min. 500 μl of cell free supernatant, 50 μl of 0.1 mM orthophosphoric acid, and 2 ml of Salkowski reagent (which contains 1 ml of 0.5 M FeCl_3_ dissolved in 50 ml of 35% perchloric acid) were added. After that, the mixture was placed in the dark for 30 minutes to allow for the development of color. It was considered that the pink-to-red color that resulted from exposure to the Salkowski reagent was an indication that the bacterium produced indole acetic acid. Color intensity was measured spectrophotometrically (M/s. Shimadzu, Japan) at 530 nm. IAA production was calculated using the standard curve and expressed as µg ml^-1^. In order to measure the 1-aminocyclopropane-1-carboxylate (ACC) deaminase activity, the bacterium was cultured in DF minimum salt medium with 3 mM ACC added as the only source of nitrogen supply, at different salt concentrations (0%, 7% w/v NaCl), and 30% PEG 6000. By comparing the ACC deaminase activity of bacterial isolate with the α-ketobutyrate standard curve at 540 nm, quantitative measurements were made, and the results were reported as nmol of α-ketobutyrate released min^-1^ mg^-1^ of protein [20]. Biofilm formation assay was done in a 96-well microtiter plate. To identify the performance of S23 under drought and salt stress conditions, broth with 0%, 7% NaCl, and 30% PEG is used. In the 96-well microtiter plate, 10 µl of 24 hours old culture (1x 10^7^ CFU /ml) was inoculated in 150 µl of nutrient broth. The bacterium was allowed for 2 days of incubation in the microtiter plate. Then the plate was washed with distilled water 2-3 times and allowed to dry. 150 µl of 1% crystal violet stain was added and waited for 45 min for adherence. The plate was washed 2-3 times with sterile distilled water. The formation of purple rings on the edge of the well indicated the biofilm formation ability. For quantitative analysis, 200 µl of 95% ethanol was added to the wells and distained the crystal violet. Observed the color intensity of the solution in wells at 590 nm using a microtiter plate reader [21]. Extraction of exopolysaccharide (EPS) was done by using a 3-day-old bacterium cultured in a liquid nutrient medium amended with 30% PEG, 0%, and 7% NaCl. The cell-free supernatant was collected through centrifugation at 10000rpm for 10 min. The cell-free supernatant and ethanol (90%) were added in the ratio of 1:2 and incubated at -20ºC for 24 hours to precipitate EPS. Then the precipitate containing crude EPS was dissolved with 2ml of water. After that, 1 ml of 93% sulfuric acid and 200 µl of 5% phenol were added and incubated at room temperature for 10 minutes. The formation of yellow color was the indication of EPS production. The color intensity was observed at 490 nm using a spectrophotometer. A standard curve was drawn by using different concentrations of glucose from the 10ppm stock solution [22]. Siderophore production was quantitatively estimated by a supernatant of 48 hours old bacterial culture grown in a liquid nutrient medium (with and without stress) [23]. The supernatant was collected through centrifugation at 10,000 rpm for 10 min. Then 0.5 ml supernatant was mixed with 0.5 ml CAS (Chromazural S) reagent and incubated for 20 min then optical density was measured at 630 nm. Siderophore production was expressed as percent siderophore unit (psu)and calculated as


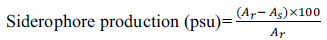


Where, *A_r_*= absorbance of CAS solution and uninoculated broth (reference)

*A_s_*= absorbance of CAS solution and cell-free supernatant of sample (sample)

Solubilization of insoluble phosphate was quantitatively estimated according to Watanabe and Olsen [24]. The bacterium was inoculated in 10 ml of Pikovskaya’s broth supplemented with 0.5% tricalcium phosphate with and without PEG (30%), NaCl (7%), and incubated for 5 days at 28 ± 2°C then the supernatant was collected by centrifugation at 10,000 rpm for 5min. 1 ml of supernatant and 0.8 ml of color reagent were added to a test tube, and the volume was made up to 5 ml with distilled water. After 15 minutes at room temperature, the color developed was measured with a spectrophotometer at 660 nm. KH_2_PO_4_ was used for standard curve preparation [24]. Estimation of osmolytes accumulation in the endophytic bacterium *E*. *cloacae* S23's proline, glycine betaine, and trehalose concentrations were estimated. Liquid nutrient medium with 0%, 7% (w/v) NaCl concentration and drought condition (-10.7 bars) by using 30% Polyethylene glycol PEG 6000 to maintain normal, salt-stressed, and moisture-stressed conditions, respectively. 1 percent of *E*. *cloacae* S23 (1x 10^7^ CFU/ml) was inoculated and incubated for 24 hours at 28 ± 2°C with shaking. To measure proline, cells were extracted by centrifugation at 10,000 rpm for 10 minutes. The cell pellet was boiled in 80% ethanol in a water bath at 60°C for 45 minutes, after which the suspension was centrifuged at 8000 rpm for 15 minutes, and 1 ml of supernatant was collected. Then, 1 ml of acid ninhydrin and 1 ml of glacial acetic acid were added to the supernatant. The reaction mixture was maintained in 100°C boiling water for one hour before being transferred to the ice for cooling. To extract proline from the reaction mixture, 2 ml of toluene was added. A spectrophotometer (M/s. Shimadzu, Japan) was used to measure the absorbance at 520 nm for the extracted proline, which was pinkish to red. The proline was used to create a standard curve [25]. The result is expressed as micrograms of proline per milliliter of bacterial culture. To estimate glycine betaine, cells were extracted by centrifugation at 10,000 rpm for 10 minutes and then diluted 1:1 with 2 N sulfuric acid. In the test tube, an aliquot of 0.5 ml was collected and chilled in ice water for one hour. The liquid was gently vortexed after the addition of 0.2 ml of a cold iodine reagent. After 16 hours at 4 °C, the sample was placed in a centrifuge tube and centrifuged at 0°C at 10,000 rpm for 15 minutes. The supernatant was removed carefully using a 1 ml micropipette, and the centrifuge tube was placed on ice. In 9 mL of 1, 2-dichloroethane, the precipitate was dissolved and thoroughly mixed. The absorbance at 365 nm was measured after 2.0-2.5 hours using a spectrophotometer (M/s. Shimadzu, Japan). 50-200 mg ml^-1^ glycine betaine standards were prepared in 2N sulfuric acid [26]. The result is expressed as g of glycine betaine per milliliter of bacterial culture. For trehalose measurement, the cell pellet was collected by centrifugation at 10,000 rpm for 10 minutes, and trehalose was extracted from the pellet by overnight incubation in 70% (w/v) ethanol. After incubation, cell debris was removed by centrifugation at 10,000 rpm for 10 minutes, then the supernatant devoid of cells was dried at 70°C. In 10 ml of distilled water, the residue was dissolved. 1 ml of the aliquot was transferred to a test tube, followed by the addition of 2 ml of anthrone reagent (200 mg l^-1^ anthrone in 95% sulfuric acid) and 15 minutes of incubation in a boiling water bath. After incubation, the assay mixture was chilled on ice for 5 minutes before absorbance measurement at 630 nm. Trehalose concentration was determined using a standard curve consisting of 0-50 g m^l-1^ trehalose.

## 3 RESULTS

### 3.1 Whole-genome Sequencing and Assembly

The whole-genome sequencing of the genome of S23 was performed using the Illumina-Miseq platform. 99.3% of coarse consistency and 98.6% of fine consistency were obtained, indicating the genome is more self-consistent which confirmed that our genome quality is good. The estimated length of the genome is 4,824,008 base pairs, and the sequencing reads were assembled into 35 contigs. The L50 count, which is the smallest number of contigs whose length sum generates the N50, is 4, and the N50 length, which is the shortest sequence length at 50% of the genome, is 475,203 bp. The phylogenetic analysis derived from the comparison of protein-coding genes indicated that S23 is a member of the Enterobacteriaceae family and its genome size is similar to those found in other members of their family. The GC content of the S23 isolate is 55.10%. *E*. *cloacae* S23 belongs to phylum Proteobacteria of class Gammaproteobacteria, within the order Enterobacterales of the Enterobacteriaceae family. Furthermore, the genome of S23 comprised specialty genes (Table **S1**), including antibiotic resistance-58 (Source, CARD), antibiotic resistance – 4 (source, NDARO) antibiotic resistance-62 (Source, PATRIC), transporter-574 (Source, TCDB), drug target-305 (Source, Drug Bank) and drug target-55(Source, TTD).

### 3.2 Gene Prediction and Annotation

**Fig. (1):**
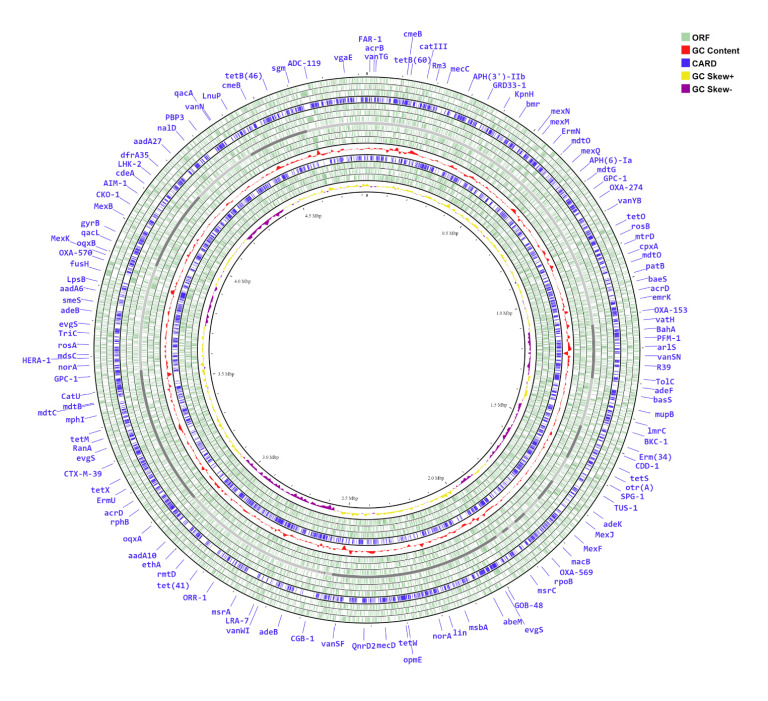
Circular Graphical Assembly of the genome of *Enterobacter cloacae* S23 isolate. From the outer circle to the inner circle: tRNA operons, open reading frames (ORF’s), and rRNA are shown in green. GC content contents are plotted in the genome in red color. map where GC skew (yellow: positive values and violet: negative values) and CARD (Comprehensive Antibiotic Resistance Database) are shown in blue color.

RAST tool kit (RASTtk) was used to annotate the genome of *E*. *cloacae* S23, and a unique genome identifier of 1005665.10 was assigned. Annotations from *E*. *cloacae* S23 confirmed that this genome contained 4511 protein-coding sequences (CDS), 71 transfer RNA (tRNA) genes, and 3 ribosomal RNA (rRNA) genes along with 23 repeated regions. The genome annotation of S23 also revealed that 4,421 CDS (28%) were assigned to the cross-genus protein families (PGFams) whereas 3510 CDS (22%) were treated as genus-specific protein families (PLFams). The CDS with functional assignments included 1,272 (8%) proteins with Enzyme Commission (EC) numbers, 1,039 (7%) with Gene Ontology (GO) assignments, and 905 (6%) proteins that were mapped to KEGG pathways. For the CDSs with unassigned functions, 580 CDSs (4%) were recognized as conserved hypothetical proteins. GC Skew value of *E*. *cloacae* S23 was calculated using skewIT online tool. The results show the GC skew value (skewl= 0.125). The distribution of genomic annotations is shown in a circular graph. From the outer circle to the inner circle: tRNA operons, open reading frames (ORFs), and rRNA are shown in green. GC contents are plotted in the genome in red color. In a circular map where GC skew (yellow: positive values and violet: negative values) and CARD (Comprehensive Antibiotic Resistance Database) are shown in blue color (Fig. **1**).

### 3.3 Genome Comparison

**Fig. (2):**
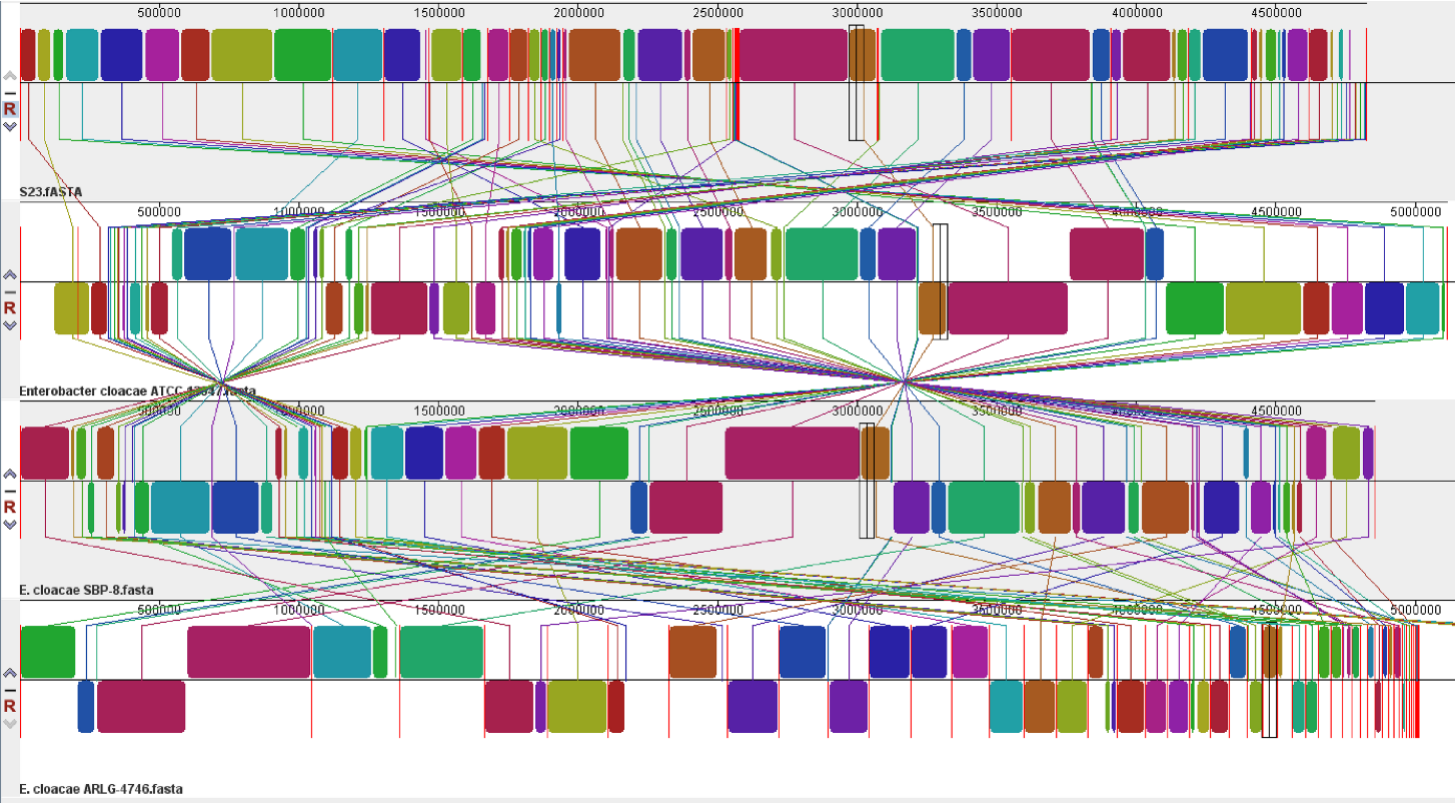
Global multiple alignments of chromosomes from four closely related *Enterobacter* strains using progressive Mauve with default parameters. Colored blocks indicated the genome sequences that aligned to part of another genome and were possibly homologous and internally free from genomic rearrangement (locally collinear blocks - LCB). White regions represented sequences that did not align and probably contained sequences specific to a particular genome. Blocks below the center line showed regions that aligned in the reverse orientation. The names of the strains were listed at top of the blocks.

**Fig. (3):**
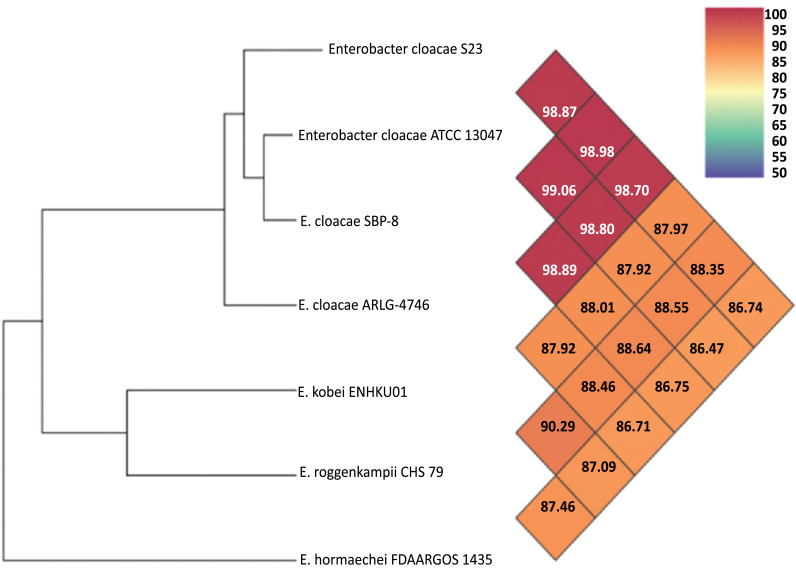
Phylogenetic tree based on the Whole genome sequence of the *Enterobacter cloacae* S23 and other strains belonging to the genus *Enterobacter*. The scale bar represents the distance corresponding to 0.04 changes per nucleotide position.

We selected the whole sequenced genomes of four *Enterobacter* strains (*Enterobacter cloacae* S23, *Enterobacter cloacae* ATCC 13047, *Enterobacter cloacae* SPB-8, and *Enterobacter cloacae* ARLG-4746, isolated from different habitats for comparative genomic analysis. Each colored block represents a region of sequence that aligns with part of another genome and is presumably homologous and free from internal rearrangements. These are called LCBs (locally collinear blocks). Using the software Mauve, a global alignment of genome sequences from these four *Enterobacter* strains was performed, and the results demonstrated the significant similarity between the *E. cloacae* S23 genome, with only a few instances of genome rearrangement (Fig. **2**). Table **S4** displays the 23 *E. cloacae* species closely related to *E. cloacae* ATCC 13047 (98.86), *E. cloacae* SBP-8 (98.97), and *E*. *cloacae* ARLG-4746 (98.69) based on the results of the orthoANI analysis [27]. Compared to *E*. *cloacae* S23, *Enterobacter hormaechei* FDAARGOS 1435 has a lower threshold value (86.74). The findings show that the S23 belongs to *E*. *cloacae* (Fig. **3**).

### 3.4 Phylogenetic Analysis

**Fig. (4):**
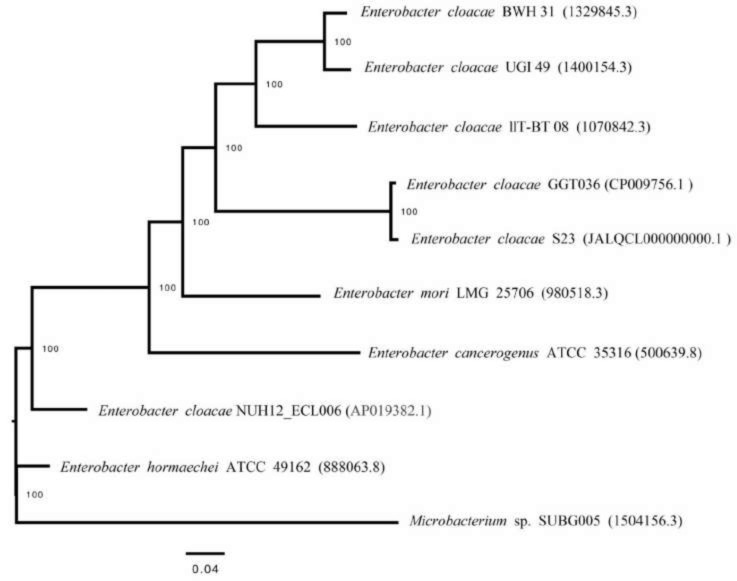
Subsystem Analysis of the Genome *Enterobacter cloacae* S23.

The phylogenetic tree indicated the S23 formed a cluster with *Enterobacter cloacae* GGT036550. Similarly, *Enterobacter cloacae* BWH31 strain formed a separate cluster with *Enterobacter cloacae* UCI 491400154. *E*. *hormaechei* strain ATCC 49162 formed a separate cluster. The phylogenetic tree revealed that *Enterobacter cloacae* are polyphyletic which is clustered with other *Enterobacter* members in a different position. The out-group *Microbacterium* sp. SUBG005 formed a separate clade and deep phylogenetic lineage compared to other *Enterobacter* strains. Similarly, *Enterobacter cloacae* NUH12_ECL006 550.1741 was placed in a separate clade from other *Enterobacter* spp. (Fig. **4**).

### 3.5 Plant Colonization and Adhesion

Gene annotation of the S23 genome revealed that it contains genes responsible for colonization in the exosphere (host factor I protein), endosphere (carbamoyl phosphate synthase), and others (*fts, pil*) (Table **1**) colanic acid synthesis (wca, wzc, wza, *wcal*), chemotaxis (*che*) and curlin genes accounted for plant-microbe adhesion (Table **S2**).

### 3.6 Virulence Factor

The virulence factor of *E*. *cloacae* S23 was analyzed and compared with three different sources. The isolate S23 has 148 (Source- Victors), 119 (PATRIC_VF), and 25 (VFDB) genes responsible for virulence factors. Out of 148 genes for VF, only 10 genes have query coverage of 100% and identity percent of >98%. Of the selected 10 genes, 5 genes are involved in stress management (Table **2**).

### 3.7 Antimicrobial Resistance

PATRIC used a k-mer-based AMR genes detection approach to detect the presence of antimicrobial resistance genes (AMR) in *E*. *cloacae* S23. Antimicrobial resistance is caused by a total of 62 genes, which are classified into ten main mechanisms of action. Their mode of action mainly depends on gene activation and environmental condition (Table **3**).

### 3.8 Functional Analysis of Genome

**Fig. (5):**
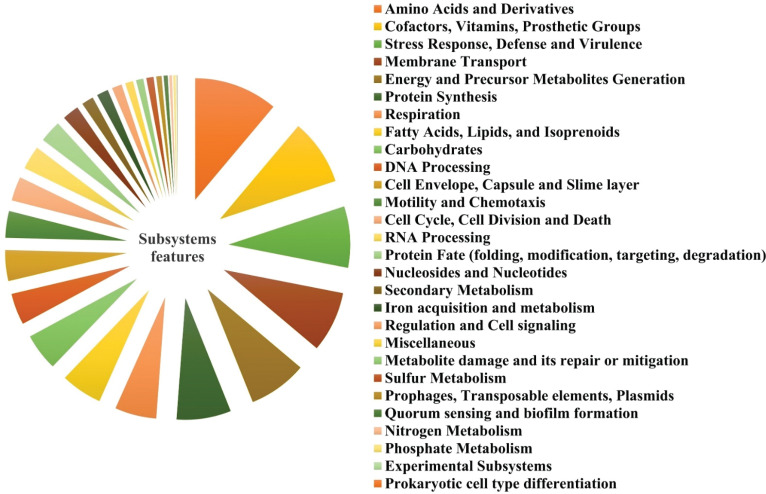
Subsystem analysis of the genome *Enterobacter cloacae* S23.

Gene ontology (GO) and KEGG pathways were used to perform functional analyses on the annotated genomes of *E*. *cloacae* S23. This revealed that the occurrence of 266 (11.12%), 210 (8.7%), 195 (8.15%), 193 (8.07%),186 (7.7%), 173 (7.23%), 132 (5.5%), (131 (5.48%), 118 (4.9%), 118 (4.9%), 100 (4.18%) and 98 (4.10%) genes involved in the metabolism of amino acids and derivatives, cofactors, vitamins, and prosthetic groups, stress response, defense and virulence, membrane transport, energy, and precursor metabolites generation, protein synthesis, respiration, fatty acids, lipids, and isoprenoids, carbohydrates, DNA processing, and cell envelope, capsule, and slime layer as the major contribution. The remaining metabolisms are contributed at a minor level (<4%) (Fig. **5**).

### 3.9 Plant Growth Promotion

*Enterobacter cloacae* S23's genome contains genes for nutrient solubilization (kdp, pho, pst), biofilm formation (pga), IAA production (trp), and siderophore production (luc, fhu, fep, ent, ybd) (Table **S3**). The plant growth promoting activities of *E*. *cloacae* S23 were evaluated under normal, 7 percent salt (salt stress), and 30 percent PEG conditions (drought stress). Initially, the stress tolerance of an isolate was determined by allowing it to grow in a liquid nutrient medium supplemented with 7% salt and 30% PEG; this results in the isolate's survival under the stress condition. Under stress conditions, the amount of IAA produced was lower than under normal conditions, but isolate S23 was able to produce IAA under both salinity and drought stress. IAA is one of the essential plant growth hormones that regulate plant development and promote root development. Under stressful conditions, *E*. *cloacae* S23 produced more exopolysaccharides (EPS) than under normal conditions. Under stress conditions, ACC deaminase activity was greatly increased; this decreases ethylene production in the host and delays senescence. During stressful conditions, siderophore production increases, indicating that the S23 has a greater capacity for iron sequestration, which starves pathogens for iron. The isolate S23 is also capable of forming a robust biofilm under normal and stressful conditions. Phosphorus measurement revealed that phosphate solubilization ability was marginally enhanced under stress conditions. The S23 isolate was exposed to osmolyte accumulation under both normal and stress conditions, and the proline (stress indicator) content increased under stress (Table **4**).

## 4 DISCUSSION

In the present study, whole genome sequecinng of *Enterobacter cloacae* S23 obtained through Illumina-Miseq platform resulted in 35 conigs. Assembly of short reads from technologies such as Illumina often yielded assemblies with a large number of contigs however 50 contigs per genome is acceptable [28]. In the current study number of contigs generated for *E*. *cloacae* S23 is with in acceptable limit as stated by Theo [28]. In *E*. *cloacae* S23, 5 virulence factor genes are responsible for stress management. The *ghmA* (Sedoheptulose 7-phosphate isomerase) gene catalyses the first committed step in the heptose biosynthesis of the inner core component of lipopolysaccharide *i.e*., the isomerisation of D-sedoheptulose 7-phosphate to D-glycero-D-manno-heptose 7-phosphate [29]. This helps in lipid formation which is one of the major stress tolerance mechanisms in microbes in adverse conditions. Cold shock proteins (CSP) mainly aid in surviving cold shock. But some CSP helps in the regulation of heat shock protein (HSP), as overexpression of the cold shock proteins *CspC* and *CspE* causes a threefold rise in two of the key Hsps—*DnaK* and *GroEL* [30]. The heat shock proteins help microbes to survive drought conditions and high temperatures. The *Ion* protease is involved in iron-dependent oxidative stress response pathways and is also involved in acid stress control [31]. Thioredoxins are involved in the elimination of harmful disulfides that might have been generated in cytosolic proteins as a result of oxidative stress [32]. When exposed to hyperosmotic stress, *OmpR* facilitated the inducible transcription of the *ompC*, *F*, and *X* genes [33]. These genes of virulence factors helped the S23 to withstand various stress conditions. Functional annotation of the S23 genome revealed that a total of 2390 genes were involved in the biological, molecular, and cellular component system. Metabolism of amino acids and derivatives, cofactors, vitamins, and prosthetic groups, stress response, defense and virulence, membrane transport, energy and precursor metabolites generation, protein synthesis, respiration, fatty acids, and lipids are the top 10 predominant biological and molecular functions. These components contributed to a functional group of genes that help plants thrive. The annotated genes were then organized into one or more subsystems and designated functional roles. 2390 genes of the annotated genome of S23 are classified into 28 functional categories. Some proteins are found to be responsible for drought and salinity stress tolerance including amino acid derivatives that contain Osmo regulation proline transporters, multiple antibiotic resistance (*i.e*., triclosan resistance, fusidic acid resistance, bicyclomycin resistance cluster, fosfomycin resistance, polymyxin resistance, mupirocin resistance, daptomycin resistance, arsenic resistance, bacitracin resistance, and fusaric acid resistance cluster), and also have heat shock dnaK gene cluster extended and cold shock proteins of CSP family. The ANI values obtained for S23 with other *E*. *cloacae* including type strain were more than the threshold value of 98%. Hence, the ANI result indicated that S23 belongs to *E*. *cloacae*. The phylogenetic tree constructed with the whole genome of *Enterobacter* revealed that *Enterobacter cloacae* S23 cluster with *E*. *cloacae* GGT036 and supported with 100% bootstrap value. *Enterobacter cloacae* NUH12_ECL006 (SAMD00143514) was placed in a separate clade from other *Enterobacter* spp. Carbapenem-resistant *Enterobacter cloacae* NUH12_ECL006 was isolated from a human in Japan by Tetsuka *et al*. [34] and it formed a separate clade from other *Enterobacter* isolated from soil and plants. Previously *Pseudomonas aeruginosa* isolated from clinical specimens formed a separate clade with environmental isolates [35]. Some of the *Enterobacter* species are beneficial endophytes. Endophytes colonize in the host cell utilizing natural opening, damaged tissue, or through cell wall hydrolytic enzymes [36]. The genome S23 showed the colonic acid synthesis gene which was the critical element for biofilm and exopolysaccharide production that is involved in the adhesion of microbes to the host. Along with colonic acid S23 genome contains curlin gene and chemotaxis genes, that aid in the host-bacterium interaction [37]. Endophyte colonization includes genes mostly involved in the recognition of the host plant [38]. The gene annotation of the S23 genome also accounts to plant- microbe interaction and colonization (Supplementary material Tables **S1** and **S2**). The functional analysis resulted in many genes of functional class helping in plant growth and adaptation strategies during environmental stress conditions. *E*. *cloacae* S23 isolate is active in drought conditions and performs its beneficial role even in stressed conditions. Growth analysis and survivability test showed S23 survived up to -10.7 bars of water potential. Further activities were analyzed at -10.7 bars to understand their function during stress conditions. Estimated IAA was low both in a stressed and unstressed condition, genomic analysis revealed that it has 6-8 genes responsible for the tryptophan biosynthesis which can be utilized in various processes. EPS production is the major stress tolerance mechanism, as they protect them from desiccation effects and nutrient recycling. One of the common methods of adaptation to stress condition is EPS production and biofilm-forming ability, S23 have greater EPS production and strong biofilm-formation capacity. This is also justified by functional protein classes’ *i.e*., fatty acid and lipid productions are one the major functional groups with 131 active genes. Insoluble phosphate solubilization capacity plays a prominent role; phosphorous is the key element in many physiological processes such as photosynthesis, cell division, and root development. Solubilisation of insoluble phosphate by the isolate S23 increases the nutrient uptake of the host. Apart from these characteristics, *E*. *cloacae* S23 also has the ability for the accumulation of osmolytes under drought stress as the adaptive mechanism. Siderophore production is widely utilized by endophytes for ion acquisition and is also a major PGP trait. *E*. *cloacae* S23 also can secrete siderophore in normal and stressed conditions. Rhizosphere soil is a complex ecological system, with a large range of hosts and the environmental condition that influence the plant-microbe interaction and microbial community. The genome of *Enterobacter cloacae* S23 contains genes for nutrient solubilization, biofilm formation, IAA production, and siderophore production. By interpreting the result obtained by biochemical and molecular analysis, it was evident that *Enterobacter cloacae* (S23) have plant growth-promoting traits (Table **S3**). Drought screening of non-rhizobial passenger endophytes *E*. *cloacae* S23 exhibits beneficial action to the host even under severe drought stress (*i.e*. -10.7bars). Drought tolerance capacity and its PGP activities under *in vitro* conditions revealed that the *E*. *cloacae* S23 is probably a good colonizer in the rhizosphere soil during stress conditions. It is expected that the microbial community's survival and activity are influenced by the chemical characteristics and content of root exudates. However, the enrichment of *E*. *cloacae* S23 in the peanut rhizosphere region ensures plant survival during adverse conditions.

## CONCLUSION

The whole genome of *E*. *cloacae* S23 offers suggestions for understanding the molecular mechanisms governing plant growth promotion and abiotic stress tolerance, and it may help create entophytic bacteria as inoculants to increase agricultural production in marginal and arid regions.

## Figures and Tables

**Table 1:** Annotation of genes responsible for plant colonization.

**Class**	**Function**	**Gene and its Product**
I Exosphere	Global regulator	Host factor – I protein
	Hfl operon	SSU_ribosomal_protein_S1p
		Putative_inner_membrane_protein_YjeT_(clustered_with_HflC)
		RNA-binding_protein_Hfq
		HflC_protein
		HflK_protein
	Nutritional adaptation	Phospho-N-acetylmuramoyl-pentapeptide-transferase_(EC_2.7.8.13)
II Endosphere	DSF-degrading enzyme	Carbamoyl-phosphate_synthase_large_chain_(EC_6.3.5.5)
		Carbamoyl-phosphate_synthase_small_chain_(EC_6.3.5.5)
III Colonization	FtsEX cell-division-associated signaling system	Signal_recognition_particle_receptor_FtsY
	Type IV pilus	Type_IV_fimbrial_assembly_protein_PilC
		Type_IV_pilus_biogenesis_protein_PilM
		Twitching_motility_protein_PilT
		Type_IV_pilin_PilA
		Type_IV_fimbrial_assembly,_ATPase_PilB
DSF – diffusible signal factor		-

**Table 2:** Genes of virulence factor responsible for stress response.

**Gene**	**Product**	**Query Coverage**	**Identity Percentage**
gmhA	D-sedoheptulose 7-phosphate isomerase	100	98
cspC	Cold shock protein of the CSP family	100	100
Ion	ATP-dependent protease La	100	98
trxA	Thioredoxin	100	100
ompR	Two-component system response regulator OmpR	100	100

**Table 3:** Major mechanisms involved in antimicrobial resistance.

**AMR Mechanisms**	**Gene Count**	**Genes Responsible**
Antibiotic activation enzyme	1	KatG
Antibiotic inactivation enzyme	1	CMH family
Antibiotic resistance gene cluster, cassette, or operon	3	MarR, MarA, MarB
Antibiotic target protection protein	1	BcrC
Efflux pump conferring antibiotic resistance	18	SugE, MdtL, EmrAB-TolC, AcrEF-TolC, MdtABC-TolC(2), TolC/OpmH,
Gene conferring resistance *via* absence	1	gidB
Protein altering cell wall charge conferring antibiotic resistance	3	GdpD, GdpD, PgsA
Protein modulating permeability to antibiotic	1	OccD6/OprQ
Regulator modulating the expression of antibiotic resistance genes	4	AcrAB-TolC, EmrAB-TolC, OxyR, H-NS
Antibiotic target in susceptible species	29	EF-Tu(4), Ddl(2), folA, Df(2)r, dxr, EF-G(2), gyrB, Alr(2), rpoC, kasA(4), S12p,rpoB, gyrA, folP, S10p, rho, Iso-tRNA, MurA, inhA, fabI

**Table 4:** Plant growth promoting potential of *Enterobacter cloacae* S23 under stressed conditions.

**Plant Growth Promoting Traits**	**Without Stress**	**With Moisture Stress** **(-10.7 bars)**	**Salt Stress** **(7% NaCl)**
Growth (OD@660nm after 24hrs)	0.502	0.102	0.203
IAA estimation (µg/ml)	0.52	0.30	0.37
EPS production (µg/ml)	22.9	98.4	89.4
*Biofilm forming capacity	strong	moderate	strong
Proline accumulation (µg/ml)	7.03	17.73	18.21
Trehalose accumulation (µg/ml)	204.3	190.58	189.24
Glycine beatine accumulation (µg/ml)	22	16.2	17.51
Phosphorous quantification (µg/ml)	0.26	1.43	1.09
Siderophore production (psu)	2.29	23.26	19.56
ACC deaminase activity (nmol α-ketobutyrate released min-^1^) mg^-1)^ protein)	7.00	12.99	14.26

Note: *For biofilm formation Strong – OD value >0.3; Moderate – OD value ranges from 0.2-0.3; Negative – OD value < 0.1

## Data Availability

*Enterobacter cloacae* strain S23, whole-genome shotgun sequencing project data have been deposited at GenBank under the accession number PRJNA832885. The assembled contigs are available at https://www.ncbi.nlm.nih.gov/  assembly: GCA_023241695.1 repository with all the annotations details in the Readme file. The data generated and analyzed during this study are included in this article and its supplementary information files.

## LIST OF ABBREVIATIONS

ACC–1: aminocyclopropane-1-carboxylate
CAS: Chrome Azurol S
CDS: protein coding sequence
DF: Dworkin and Foster
GC: Guanine-cytosine
hrs: hours
KEGG: Kyoto Encyclopedia of Genes and Genomes
nm: nanometer
PEG: Polyethylene glycol
ppm: parts per million
psu: siderophore production unit
µg: microgram
µl: microlitre

## References

[1] Glick B.R. (2012). Plant growth-promoting bacteria: Mechanisms and applications.. Scientifica.

[2] Azua-Bustos A., González-Silva C. (2014). Biotechnological applications derived from microorganisms of the Atacama Desert.. BioMed Res. Int..

[3] Benslama O., Boulahrouf A. (2013). Impact of glyphosate application on the microbial activity of two Algerian soils.. Int. J. Curr. Microbiol. Appl. Sci..

[4] Delgado M., Mendez J., Rodríguez-Herrera R., Aguilar C.N., Cruz-Hernández M., Balagurusamy N. (2014). Characterization of phosphate-solubilizing bacteria isolated from the arid soils of a semi-desert region of north-east Mexico.. Biol. Agric. Hortic..

[5] Askalany M.M., Diab M., Abdalla F.A., Hassan A.M. (2017). Studies on the effect of natural treatment on sewage water, El-Salhiya-Qena City, Egypt.. Int. J..

[6] Oulebsir-Mohandkaci H., Khemili-Talbi S., Benzina F., Halouane F. (2015). Isolation and identification of entomopathogenic bacteria from Algerian desert soil and their effects against the migratory locust, Locusta migratoria (Linnaeus, 1758) (Orthoptera: Acrididae).. Egypt. J. Biol. Pest Control.

[7] Preyanga R., Anandham R., Krishnamoorthy R., Senthilkumar M., Gopal N.O., Vellaikumar A., Meena S. (2021). Groundnut (Arachis hypogaea) nodule Rhizobium and passenger endophytic bacterial cultivable diversity and their impact on plant growth promotion.. Rhizosphere.

[8] Vimal S.R., Singh J.S., Arora N.K., Singh S. (2017). Soil-plant-microbe interactions in stressed agriculture management: A review.. Pedosphere.

[9] Coulson T.J.D., Patten C.L. (2015). Complete genome sequence of Enterobacter cloacae UW5, a rhizobacterium capable of high levels of indole-3-acetic acid production.. Genome Announc..

[10] Peng G., Zhang W., Luo H., Xie H., Lai W., Tan Z. (2009). Enterobacter oryzae sp. nov., a nitrogen-fixing bacterium isolated from the wild rice species Oryza latifolia.. Int. J. Syst. Evol. Microbiol..

[11] Soares G.G., Costa J.F., Melo F.B.S., Mola R., Balbino T.C.L. (2016). Biofilm production and resistance profile of Enterobacter sp. strains isolated from pressure ulcers in Petrolina, Pernambuco, Brazil.. J. Bras. Patol. Med. Lab..

[12] Palaniappan P., Chauhan P.S., Saravanan V.S., Anandham R., Sa T. (2010). Isolation and characterization of plant growth promoting endophytic bacterial isolates from root nodule of Lespedeza sp.. Biol. Fertil. Soils.

[13] Sturz A.V., Christie B.R., Matheson B.G., Nowak J. (1997). Biodiversity of endophytic bacteria which colonize red clover nodules, roots, stems and foliage and their influence on host growth.. Biol. Fertil. Soils.

[14] Saharan B.S., Nehra V. (2011). Plant growth promoting rhizobacteria: A critical review.. Life Sci Med Res.

[15] Schomburg I., Chang A., Ebeling C., Gremse M., Heldt C., Huhn G., Schomburg D. (2004). BRENDA, the enzyme database: Updates and major new developments.. Nucleic Acids Res..

[16] Ashburner M., Ball C.A., Blake J.A., Botstein D., Butler H., Cherry J.M., Davis A.P., Dolinski K., Dwight S.S., Eppig J.T., Harris M.A., Hill D.P., Issel-Tarver L., Kasarskis A., Lewis S., Matese J.C., Richardson J.E., Ringwald M., Rubin G.M., Sherlock G. (2000). Gene Ontology: Tool for the unification of biology.. Nat. Genet..

[17] Kanehisa M., Sato Y., Kawashima M., Furumichi M., Tanabe M. (2016). KEGG as a reference resource for gene and protein annotation.. Nucleic Acids Res..

[18] Davis J.J., Gerdes S., Olsen G.J., Olson R., Pusch G.D., Shukla M., Vonstein V., Wattam A.R., Yoo H. (2016). PATtyFams: Protein families for the microbial genomes in the PATRIC database.. Front. Microbiol..

[19] Patten C.L., Glick B.R. (2002). Role of Pseudomonas putida indoleacetic acid in development of the host plant root system.. Appl. Environ. Microbiol..

[20] Penrose D.M., Glick B.R. (2003). Methods for isolating and characterizing ACC deaminase-containing plant growth-promoting rhizobacteria.. Physiol. Plant..

[21] Djordjevic D., Wiedmann M., McLandsborough L.A. (2002). Microtiter plate assay for assessment of Listeria monocytogenes biofilm formation.. Appl. Environ. Microbiol..

[22] DuBois M., Gilles K.A., Hamilton J.K., Rebers P.A., Smith F. (1956). Colorimetric method for determination of sugars and related substances.. Anal. Chem..

[23] Arora N. K., Verma M. (2017). Modified microplate method for rapid and efficient estimation of siderophore produced by bacteria.. 3 BiotechM.

[24] Watanabe F.S., Olsen S.R. (1965). Test of an ascorbic acid method for determining phosphorus in water and NaHCO3 extracts from soil.. Soil Sci. Soc. Am. J..

[25] Ceylan S., Yilan G., Akbulut B.S., Poli A., Kazan D. (2012). Interplay of adaptive capabilities of Halomonas sp. AAD12 under salt stress.. J. Biosci. Bioeng..

[26] Qurashi A.W., Sabri A.N. (2013). Osmolyte accumulation in moderately halophilic bacteria improves salt tolerance of chickpea.. Pak. J. Bot..

[27] Han N., Qiang Y., Zhang W. (2016). ANItools web: A web tool for fast genome comparison within multiple bacterial strains.. Database.

[28] Theo H.M.S. (2019). The importance of genome sequence quality to microbial comparative genomics.. BMC Genomics.

[29] Brooke J.S., Valvano M.A. (1996). Biosynthesis of inner core lipopolysaccharide in enteric bacteria identification and characterization of a conserved phosphoheptose isomerase.. J. Biol. Chem..

[30] Cardoza E., Singh H. (2021). C group-mediated antibiotic stress mimics the cold shock response.. Curr. Microbiol..

[31] Kirthika P., Senevirathne A., Jawalagatti V., Park S., Lee J.H. (2020). Deletion of the lon gene augments expression of Salmonella Pathogenicity Island (SPI)-1 and metal ion uptake genes leading to the accumulation of bactericidal hydroxyl radicals and host pro-inflammatory cytokine-mediated rapid intracellular clearance.. Gut Microbes.

[32] Gao H., Zhang Y., Han Y., Yang L., Liu X., Guo Z., Tan Y., Huang X., Zhou D., Yang R. (2011). Phenotypic and transcriptional analysis of the osmotic regulator OmpR in Yersinia pestis.. BMC Microbiol..

[33] Collet J.F., D’Souza J.C., Jakob U., Bardwell J.C.A. (2003). Thioredoxin 2, an oxidative stress-induced protein, contains a high affinity zinc binding site.. J. Biol. Chem..

[34] Tetsuka N., Hirabayashi A., Matsumoto A., Oka K., Hara Y., Morioka H., Iguchi M. (2019). Molecular epidemiological analysis and risk factors for acquisition of carbapenemase-producing Enterobacter cloacae complex in a Japanese university hospital.. Antimicrob. Resist. Infect. Control.

[35] Radhapriya P., Ramachandran A., Anandham R., Mahalingam S. (2015). Pseudomonas aeruginosa RRALC3 enhances the biomass, nutrient and carbon contents of Pongamia pinnata seedlings in degraded forest soil.. PLoS One.

[36] Singh M., Kumar A., Singh R., Pandey K. D. (2017). Endophytic bacteria: A new source of bioactive compounds.. 3 Biotech.

[37] Mao Y., Doyle M.P., Chen J. (2006). Role of colanic acid exopolysaccharide in the survival of enterohaemorrhagic Escherichia coli O157:H7 in simulated gastrointestinal fluids.. Lett. Appl. Microbiol..

[38] Hardoim P.R., van Overbeek L.S., Elsas J.D. (2008). Properties of bacterial endophytes and their proposed role in plant growth.. Trends Microbiol..

